# Myometrial‐derived CXCL12 promotes lipopolysaccharide induced preterm labour by regulating macrophage migration, polarization and function in mice

**DOI:** 10.1111/jcmm.17252

**Published:** 2022-03-23

**Authors:** Lijuan Zhang, Ramanaiah Mamillapalli, Shutaro Habata, Molly McAdow, Hugh S. Taylor

**Affiliations:** ^1^ Department of Obstetrics, Gynecology, and Reproductive Sciences Yale School of Medicine New Haven Connecticut USA

**Keywords:** AMD3100, CXCL12, LPS, macrophage, PTL, SMC, uterus

## Abstract

Preterm birth is a major contributor to neonatal mortality and morbidity. Infection results in elevation of inflammation‐related cytokines followed by infiltration of immune cells into gestational tissue. CXCL12 levels are elevated in preterm birth indicating it may have a role in preterm labour (PTL); however, the pathophysiological correlations between CXCL12/CXCR4 signalling and premature labour are poorly understood. In this study, PTL was induced using lipopolysaccharide (LPS) in a murine model. LPS induced CXCL12 RNA and protein levels significantly and specifically in myometrium compared with controls (3‐fold and 3.5‐fold respectively). Highest levels were found just before the start of labour. LPS also enhanced the infiltration of neutrophils, macrophages and T cells, and induced macrophage M1 polarization. In vitro studies showed that condition medium from LPS‐treated primary smooth muscle cells (SMC) induced macrophage migration, M1 polarization and upregulated inflammation‐related cytokines such as interleukin (IL)‐1, IL‐6 and tumor necrosis factor alpha (TNF‐α). AMD3100 treatment in pregnant mice led to a significant decrease in the rate of PTL (70%), prolonged pregnancy duration and suppressed macrophage infiltration into gestation tissue by 2.5‐fold. Further, in‐vitro treatment of SMC by AMD3100 suppressed the macrophage migration, decreased polarization and downregulated IL‐1, IL‐6 and TNF‐α expression. LPS treatment in pregnant mice induced PTL by increasing myometrial CXCL12, which recruits immune cells that in turn produce inflammation‐related cytokines. These effects stimulated by LPS were completely reversed by AMD3100 through blocking of CXCL12/CXCR4 signalling. Thus, the CXCL12/CXCR4 axis presents an excellent target for preventing infection and inflammation‐related PTL.

## 1 INTRODUCTION

Preterm birth is a major contributor to neonatal mortality and morbidity.^1^ Inflammation, often as a result of infection, is a major aetiology of preterm birth.^2^, ^3^ Current treatment for preterm labour (PTL) focuses on optimization of the foetal status with corticosteroids to promote foetal lung maturity and magnesium for foetal neuroprotection.^4^ Betamimetic medications have been shown to stop premature contractions for short durations in order to administer corticosteroids, but do not prolong pregnancy more than a few days.^5^ Furthermore, in the setting of PTL with intact foetal membranes, empiric antibiotics have not been found to prolong pregnancies or to improve neonatal outcomes.^6^ While infections such as group B streptococcus (GBS) colonization,^7^ bacterial vaginosis^8^ and urinary tract infections^9^ are associated with PTL, the molecular and cellular mechanisms by which they induce preterm contractions are still not well understood. A more complete understanding of the molecular mechanisms underlying PTL is necessary to develop new therapeutic agents to prevent preterm birth.

Since a common cause of PTL is local or generalized infection, PTL exhibits common features whereby increased inflammatory mediators, such as tumor necrosis factor alpha (TNF‐α), interleukin (IL)‐1 and IL‐6, are observed in the peripheral blood and gestational tissue before the onset of uterine contractions.^10^, ^11^, ^12^, ^13^ These mediators induce the production of prostaglandins, which are crucially involved in inducing myometrial contraction.^14^ During PTL immune cells, such as macrophages, neutrophils and T cells abundantly infiltrate into gestational tissues, including the myometrium,^15^ placenta^16^ and decidua.^17^ These immune cells are a rich source of inflammatory cytokines.^18^

Chemokines bind to their respective G‐protein‐coupled receptors and promote migration of leukocytes during normal immune function and as a key aspect of the inflammatory response to infection.^19^ CXCL12 or stromal cell‐derived factor‐1 (SDF‐1) is a cytokine, or signalling molecule, that belongs to CXC group of chemokines and can induce directed chemotaxis in nearby responsive cells.^20^ CXCL12 is the ligand for G‐protein‐coupled receptors CXCR4 or CXCR7, which, through multiple divergent signals, direct chemotaxis,^21^, ^22^, ^23^, ^24^ inflammation,^25^ intracellular calcium release^26^ and gene transcription.^27^ AMD3100, a highly specific antagonist to the CXCR4 receptor, competitively blocks CXCR4.^28^ CXCL12/CXCR4 interaction is also important in physiological and in pathological processes during pregnancy. The CXCL12/CXCR4 axis is known to enhance cross talk at the maternal–foetal interface, thus influencing multiple processes, including trophoblast invasion,^29^ proliferation and survival,^30^ placental angiogenesis^31^ and immune tolerance.^32^ In addition, an increasing number of studies have demonstrated that the CXCL12/CXCR4 axis also has a pleiotropic role in several pregnancy‐associated diseases, such as recurrent spontaneous abortion^33^ and preeclampsia.^34^ There are very few studies looking at the role of the CXCL12/CXCR4 axis in PTL. Tseng et al. reported that higher CXCL12 levels in amniotic fluid correlated with risk of preterm delivery.^35^ Aminzadeh et al. found that the CXCL12 level in the cord blood was significantly higher in the preterm neonates, than those at term.^36^ Overall, these data emphasized that CXCL12 may be involved in PTL. However, the pathophysiological correlations between the CXCL12/CXCR4 axis and PTL are still poorly understood.

In this study, we showed that CXCL12 is upregulated during pregnancy and in response to lipopolysaccharide (LPS) treatment in a murine model. PTL was blocked by AMD3100. AMD3100 inhibited immune cells infiltration and macrophage M1 polarization in the pregnant murine uterus. Further, we demonstrated smooth muscle cells (SMC) regulate macrophage recruitment, polarization and cytokine secretion through the CXCL12/CXCR4 axis.

## 2 MATERIALS AND METHODS

### 2.1 Animals

Eight‐week‐old C57BL/6J wild‐type mice were purchased from Charles River Laboratories. Mice were housed and maintained in a room (21 ± 1°C) with a 12‐h light/dark cycle (7:00 am to 7:00 pm) with ad libitum access to food (Purina Chow; Purina Mills) and water, in the Yale Animal Resources Center (YARC) at Yale School of Medicine. All animal studies were conducted in accordance with NIH guidelines for the Care and Use of Laboratory Animals and approved by Yale University's Institutional Animal Care & Use Committee (#2016‐11589).

#### 2.1.1 Murine model for preterm birth

Lipopolysaccharide induced PTL was carried out in mice as previously described.^37^ Two female mice were co‐housed with a proven male mouse and checked for vaginal plugs the next morning as evidence of mating. The day of vaginal plug detection was designated as gestational day (GD) 0.5 of pregnancy, and the pregnant mice were separated from the males. All untreated pregnant mice delivered their pups on GD 19–21. At GD 15.5, LPS (*Escherichia coli* 0111:B4; Sigma; 25 μg in 200 μl of phosphate buffered saline [PBS]) or PBS 200 μl or LPS 25 μg ± AMD3100 (Sigma; 5 mg/kg in 200 μl of PBS) was administered intraperitoneally to pregnant mice on GD 15.5. AMD3100 was injected 30 min before LPS treatment, and repeated at the same dose 3 h later. Then, the mice were kept under constant observation until delivery. The presence of intact or partial foetal tissue in the cage was noted as the evidence for delivery, and the delivery before GD 18.5 was judged as PTL. The percentage of PTL was calculated as follows: percentage (%) = 100 × (number of LPS‐treated mice that delivered until GD 18.5/number of LPS‐treated pregnant mice).

### 2.2 Blood and tissue sampling

Mice were anaesthetized with isoflurane, and blood samples were collected from the orbital vein at GD 0.5, 15.5, 19.5 or 6 h after LPS or PBS injection, and 12 h after delivery in heparin‐containing tubes. Whole blood was centrifuged for 15 min at 1000 *g* within 30 min of collection, followed by centrifugation (10,000 *g* for 10 min at 4°C) to separate plasma and then stored at −80°C for further analysis by enzyme‐linked immunosorbent assay (ELISA). After 6 h of LPS or PBS injection, the uterus was removed, washed with PBS and longitudinally incised to remove the foetuses. Uterus, placenta and decidua were fixed in 4% paraformaldehyde for immuno‐histochemical (IHC) analysis. Randomly selected uterine implantation sites from each uterus were stored at −80°C in RNAlater or fixed in 4% paraformaldehyde for real‐time polymerase chain reaction (RT‐PCR) and Western blot analysis/IHC respectively.

### 2.3 Cell culture

The GD 15.5 mice were injected (i.p.) with 5 ml of cold RPMI‐1640, and after 5–7 min, intraperitoneal macrophages were harvested as previously described.^38^ Briefly, the mice were sacrificed and peritoneal fluid withdrawn, centrifuged (350 *g* for 5 min at 4°C), washed twice with PBS and suspended in 1 ml RPMI‐1640 (containing 10% foetal bovine serum [FBS] and 1% penicillin/ streptomycin and 1% amphotericin B). These peritoneal exudate cells were incubated at 37°C in 12‐well cell culture plates and 2 h later, non‐adherent cells were removed and the medium was replaced. The resultant cell population consisted of more than 95% macrophages based on the morphological criteria and F4/80 staining.

The uteri were removed for SMC culture. The isolated uterus was opened longitudinally, and the foetus, placenta, decidua and amnion removed. The myometrium was washed in Dulbecco's PBS, minced and then incubated in Hanks balanced salt solution containing (2‐hydroxyethyl)‐1‐piperazineethanesulfonic acid (HEPES; 25 mM), 1% penicillin/streptomycin, collagenase (1 mg/ml, 15 U/mg) and DNase (0.1 mg/ml, 1500 U/mg) for 45 min at 37°C with agitation; the tissue was pipetted gently to disperse the cells for every 15 min. Muscle cells were pelleted, washed and suspended in Ham's Dulbecco's modified Eagle medium: nutrient mixture F‐12 (DMEM/F12) (1:1) containing 10% FBS, 1% penicillin/ streptomycin and 1% amphotericin B and cultured in petri dish. Then, the cells were passaged and purified using the difference in adherence time between fibroblasts and SMC. For all experiments, SMC were used at passage 1 or 2 and were identified using anti alpha smooth muscle actin (α‐SMA).

### 2.4 Treatment of murine macrophages with primary uterine SMC conditioned media

Primary murine uterus SMC were incubated in 75mm^2^ flask with DMEM/F12 medium containing 1% FBS and 1% penicillin and at 70%–80% confluence were treated with LPS (1 ng/ml) or PBS. After 24 h, cell‐free smooth muscle cell conditioned media (SMC‐CM) was collected under sterile conditions, centrifuged at 1620 *g* to remove debris and then stored at −80°C. Macrophages were incubated with LPS‐SMC‐CM, PBS‐SMC‐CM or LPS‐SMC‐CM ± AMD3100 (25 μg/L). Macrophages were also incubated in fresh RPMI media with 1% FBS, and treated with CXCL12 recombinant protein (catalog #300‐349P; GeminiBio), LPS (1 ng/ml) or PBS alone. Total RNA was extracted from macrophages after 6 h treatment and stored at −80°C until use.

### 2.5 Quantitative real‐time polymerase chain reaction

Total RNA was isolated from uterus in TRIzol (Life Technologies) followed by purification using Qiagen cleaning kit. SMC were treated with LPS (100 ng/ml) or PBS. Macrophages were treated or incubated as described above. After 6 h, RNA was isolated by RNeasy^Ⓡ^ plus Micro kit (Qiagen). RNA (50 ng) was reverse transcribed to cDNA in a 20 μl reaction mixture using the iScript cDNA Synthesis Kit (Bio‐Rad Laboratories). Quantitative real‐time PCR was performed to evaluate gene expression using specific primers (Table 1) and SYBR Green (Bio‐Rad) optimized in the MyiQ Single Color Real‐Time PCR Detection System (Bio‐Rad). qRt‐PCR was carried out for 39 cycles of denaturation at 95°C for 10 s following activation at 95°C for 3 min, and annealing at 60°C for 30 s. The specificity of the amplified transcript and absence of primer‐dimers was confirmed by a melting curve analysis. Gene expression was normalized to GAPDH as an internal control. Relative mRNA expression was calculated using the comparative cycle threshold method (Ct), also known as 2^−ΔΔC(T)^ method. All experiments were carried out in triplicate, and nuclease‐free water was used as a negative control replacing the cDNA template.

**TABLE 1:** Primer sequences used for qRT‐PCR

Gene	Forward sequence	Reverse sequence
*CXCL12*	5′‐CGCCAAGGTCGTCGCCG‐3′	5′‐TTGGCTCTGGCGATGTGGC‐3′
*CXCR4*	5′‐AGAAGCTAAGGAGCATGACGG‐3′	5′‐GATGGGATTTCTGTATGAGGATTAGC‐3′
*Il‐1β*	5′‐CAAAAGATGAAGGGCTGC‐3′	5′‐GCTCTTGTTGATGTGCTGCTGCG‐3′
*Il‐6*	5′‐CCTCTCTGCAAGAGACTTCC‐3′	5′‐CTCCGGACTTGTGAAGTAGG‐3′
*TNF‐α*	5′‐ATGGCCCAGACCCTCACACTCA‐3′	5′‐TGGTGGTTTGCTACGACGTGGG‐3′
*TGF‐β*	5′‐GTTGAGGAAACAAGCCCAGA‐3′	5′‐GTTACCCAGGCTGGTCTCAA‐3′
*GAPDH*	5’‐TGTGTCCGTCGTGGATCTGA−3’	5’‐CCTGCTTCACCACCTTCTTGA‐3′

Abbreviation: qRT‐PCR, quantitative real‐time polymerase chain reaction.

### 2.6 Immunohistochemistry

Uterine tissue was fixed in 4% paraformaldehyde and embedded in paraffin. Five‐micrometre tissue sections were cut and stained with haematoxylin and eosin. Others were mounted on slides followed by 10 min boiling in sodium citrate (pH 6) for antigen retrieval, and blocking using 10% goat serum (Vector Laboratories) and incubated at 4°C overnight with anti‐CXCL12 primary antibody (catalog #ab18919, 1:800; Abcam), anti‐F4/80 (catalog #MA1‐91124, 1:100; Invitrogen), anti‐CD3 (catalog #ab11089, 1:200; Abcam) or anti‐N‐elastase‐neutrophils (catalog #ab68672, 1:200; Abcam) followed by 1 h at room temperature with appropriate biotinylated secondary antibody (1:200; Vector Laboratories), the signal was detected using ABC Vectastain elite reagents with DAB plus H2O2 (catalog #SK‐4105; Vector Laboratories). Tissue sections were counterstained with haematoxylin (Sigma‐Aldrich). Images of stained sections were captured using Olympus BX‐51 microscope (Olympus). The positive cells were enumerated on six randomly chosen visual fields at ×400 magnifications.

### 2.7 Immunofluorescence studies

A double‐colour immunofluorescence analysis was performed to identify the types of cells expressing F4/80 (1:100), iNOS (1:100), CD206 (1:500), CXCR4 (1:500) in mouse tissues or macrophages. Anti‐α‐SMA (1:100) and anti‐CXCL12 (1:500) were double stained in mouse muscle cells as previously described.^39^ Tissue sections on slides were incubated overnight at 4°C with pairs of following primary antibodies: anti‐CXCR4 (catalog #PA3‐305, 1:500; ThermoFisher Scientific), anti‐ anti‐CXCL12 (catalog #ab18919, 1:500; Abcam) and anti‐F4/80 (1:100; Invitrogen), iNOS (ab3523, 1:100; Abcam), CD206 (catalog #ab64693, 1:500; Abcam). Thereafter, after washing, slides were incubated with fluorochrome‐conjugated secondary Abs at room temperature for 1 h. The secondary antibodies (1:200 dilution) used were Alexa Fluor 568‐conjugated donkey anti‐goat (catalog #A11057; Invitrogen) and Alexa Fluor 488‐conjugated donkey anti‐rabbit (catalog #A21206; Invitrogen) or Alexa Fluor 647‐conjugated donkey anti‐rat (catalog #ab150155; Abcam). Sections were mounted under coverslips using Vectashield fluorescent mounting media with 4,6‐diamidino‐2‐phenylindole (catalog #H‐1200; Vector Laboratories). Visualization of the slides was performed using a laser scanning confocal microscope (Leica TCS SP5) and the CCMI LASX software. Stained cells were quantified by monitoring the average numbers of positively stained cells relative to the total number of cells from six randomly chosen fields.

### 2.8 Enzyme‐linked immunosorbent assay

Smooth muscle cells were treated with LPS (100 ng/ml) or PBS. After 24 h, the supernatant was collected, and CXCL12 levels were determined by ELISA using the Quantikine ELISA kit (catalog #DSA00; R&D Systems). Plasma from mice peripheral blood was collected, and CXCL12 was detected according to the manufacturer's recommendation.

### 2.9 Macrophage migration assay

Cell migration assays were performed in vitro in a 24‐well plates using transwell inserts with 8 μm pores (Corning) according to the method previously described with some modification.^40^ Briefly, primary macrophage cells were seeded (5 × 10^4^ cells/insert) onto the upper well of the chamber with 250 μl RPMI‐1640 medium, and the inserts were placed in 24‐well cell culture plates containing 600 μl of RPMI‐1640 medium. After starvation for 24 h, medium was removed and the lower chamber medium was changed into either: RPMI‐1640 medium with PBS; RPMI‐1640 medium with LPS (1 ng/ml); RPMI‐1640 medium with CXCL12 (100 ng/ml). Macrophages were treated with LPS‐stimulated SMC‐CM + AMD3100 (25 μg/ml) and PBS‐stimulated SMC‐CM. After 6 h incubation at 37°C in 5% CO_2_, the cells in the upper chambers were removed carefully with a wet cotton swab. The migrated cells on the bottom surface were stained with haematoxylin (catalog #R3537400‐1A; Ricca Chemical Company) and EOSIN B (catalog #SLCB3367; Sigma‐Aldrich), and the stained cells were subsequently photographed. Six evenly spaced fields of cells were counted in each well using a Olympus BX‐51 microscope at 20× magnification.

### 2.10 Statistical analysis

Data are reported as mean ± standard error of the mean. Statistical significance was set at *p* < 0.05 and determined using Prism Software (Graphpad, Inc). For normally distributed data, significance was determined using either one‐way analysis of variance for multiple comparisons or a *t*‐test. For data not normally distributed, significance was determined using a nonparametric multiple comparison test or the Wilcoxon matched‐pairs signed rank test. For enumeration data, significance was determined using Chi‐square test.

## 3 RESULTS

### 3.1 CXCL12 expression during gestation and in PTL

**FIGURE 1:**
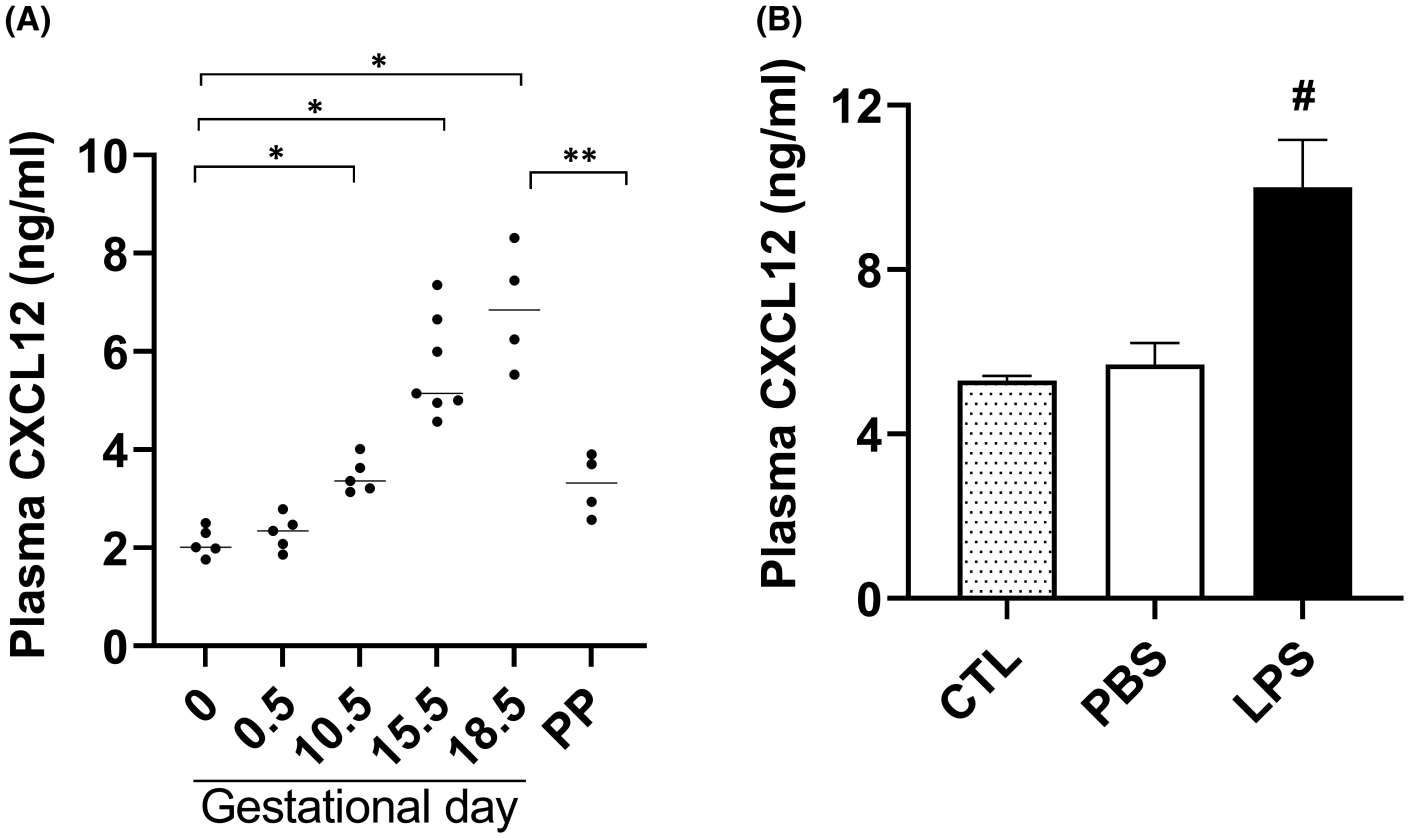
Determination of CXCL12 levels in plasma of mice by ELISA. (A) A significant increase in plasma CXCL12 levels with increasing gestational age (at time points 10.5, 15.5, 18.5 days) and a significant reduction post pregnancy (PP) compared with gestational day 18.5 while increased compared with day 0 (*n* = 6). (B) Plasma CXCL12 levels significantly increased in LPS‐treated mice compared with PBS treated or to untreated controls (*n* = 6). Each bar represents the mean ± SEM for data from two individual experiments, and each experiment was performed in triplicate. *p < 0.05 versus GD0; **p < 0.05 versus PP and **^#^** *p* < 0.05 versus PBS treated or untreated controls. ELISA, enzyme‐linked immunosorbent assay; LPS, lipopolysaccharide; PBS, phosphate buffered saline; SEM, standard error of mean

We determined the CXCL12 protein levels in the plasma collected from peripheral blood of pregnant mice using ELISA at different gestational time points (GD—GD 0.5, 10.5, 15.5, 18.5 and post pregnancy) as showed in Figure 1A. CXCL12 protein levels were significantly increased at GD 10.5, 15.5, 18.5 and at post pregnancy compared with prior to pregnancy (GD 0); protein levels were highest (3‐fold increase) just before labor (GD 18.5) and decreased significantly by 2.2‐fold after delivery (post pregnancy: pp) compared with peak CXCL12 levels. LPS treatment in pregnant mice at GD 15.5 increased the plasma CXCL12 protein levels significantly (1.5‐fold) at 6 h compared to PBS treatment as shown in Figure 1B.

**FIGURE 2:**
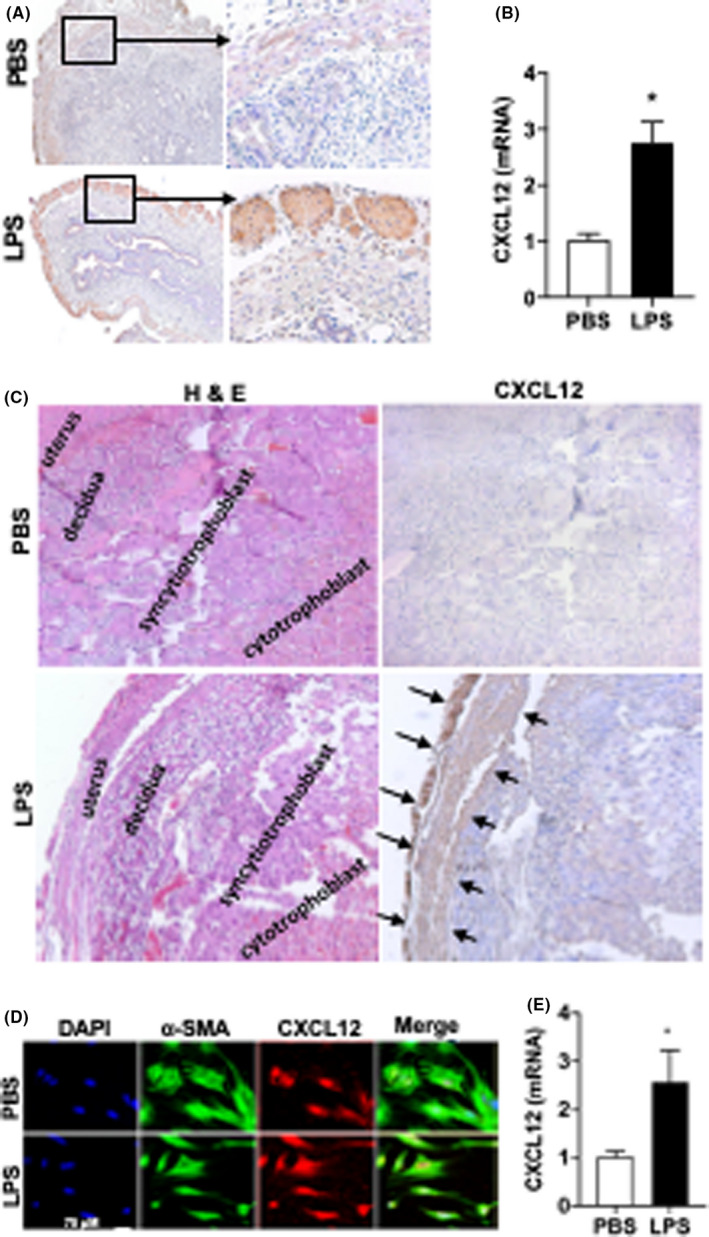
LPS upregulated CXCL12 expression in uterus. (A) Representative IHC images of CXCL12 expression. Arrow (black) shows the CXCL12 expression in myometrium. Original magnification, 100× and 400×. (B) CXCL12 mRNA levels were significantly increased in uterine tissue obtained from mice treated by LPS. Each bar represents the mean ± SEM for data from three individual experiments and each experiment was performed in duplicate. *p < 0.05 versus PBS. (C) Left: haematoxylin and eosin staining showing structural morphology of uterus, decidua, syncytiotrophoblast and cytotrophoblast treated by PBS and LPS. Right: IHC staining for CXCL12 showing representative images of uterus, decidua, syncytiotrophoblast and cytotrophoblast treated with either PBS or LPS. Original magnification (A) 100× and (B) 400×. (D, E) CXCL12 Expression in macrophages. (D) Fluorescence confocal microscopy analyses of CXCL12 expression in macrophages. Representative images of IF showing DAPI, α‐SMA, CXCL12 expression and merged images for macrophages treated with PBS and LPS. CXCL12 expression is high in LPS‐treated cells. Nuclei were stained by DAPI and are shown in blue. Scale bar: 75 µM. (E) CXCL12 mRNA levels were significantly increased in macrophages treated by LPS compared with PBS. Each bar represents the mean ± SEM for data from three individual experiments and each experiment was performed in duplicate. *p < 0.05 versus PBS. α‐SMA, alpha smooth muscle actin; DAPI, 4,6‐diamidino‐2‐phenylindole; ELISA, enzyme‐linked immunosorbent assay; IF, immunofluorescence; IHC, immunohistochemistry; LPS, lipopolysaccharide; PBS, phosphate buffered saline; SEM, standard error of mean

The increase in CXCL12 protein levels in the uterus of LPS‐treated mice was further confirmed by IHC staining as shown in Figure 2A. The greatest increase in CXCL12 was observed in the myometrium. Furthermore, Figure 2B shows a significantly increased uterine CXCL12 mRNA levels (3‐fold) after LPS treatment compared with those treated with PBS. Similarly, in pregnancy, the uterus of mice treated with LPS had higher levels of CXCL12 compared with the decidua, syncytiotrophoblasts (STBs) or cytotrophoblasts (Figure 2C).

Cultured murine uterine SMC treated with LPS showed increased expression of CXCL12 protein as shown in Figure 2D. Consistent with these findings, CXC12 mRNA levels significantly increased (2.8‐fold) in LPS‐treated cells compared with PBS treatment as shown in Figure 2E. Taken together, these results showed the upregulation of CXCL12 in response to LPS and suggested a possible role in LPS‐induced PTL.

### 3.2 AMD3100 reversed LPS effects on PTL

**FIGURE 3:**
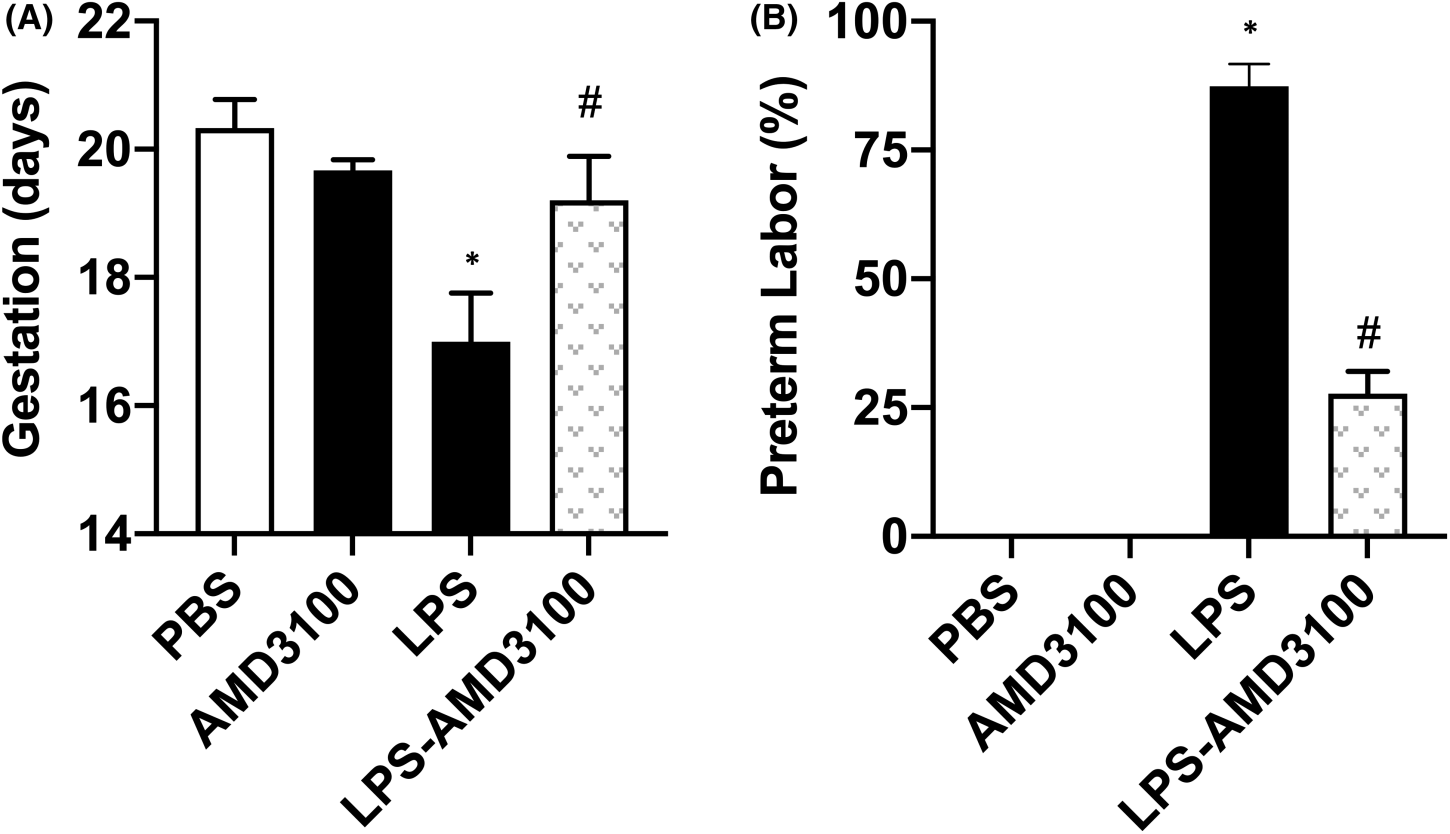
AMD3100 rescued PTL induced by LPS. (A) AMD3100 alone had no effect on the length of gestation. LPS significantly decreased gestational length and this effect was rescued by AMD3100. (B) AMD3100 significantly reduced the rate of PTL induced by LPS. Results shown as mean ± SE. *p < 0.05 versus PBS or AMD3100 while **^#^** *p* < 0.05 versus LPS. LPS, lipopolysaccharide; PBS, phosphatecbuffered saline; PTL, preterm labour; SE, standard error

In order to evaluate the role of CXCL12‐CXCR4 axis in PTL, we administered the CXCR4 antagonist AMD3100 to pregnant mice 30 min before LPS injection. Based on the half‐life of AMD3100, we repeated AMD3100 injection after 3 h. AMD3100 treatment alone (in the absence of LPS) had no effect on the length of pregnancy or the incidence of PTL. LPS treatment significantly reduced pregnancy duration by 3 days with 83% mice delivering preterm, while AMD3100 suppressed the LPS effect and significantly prolonged pregnancy duration (Figure 3A). The rate of PTL was significantly suppressed with AMD3100 treatment by 3.2‐fold in LPS‐treated pregnant mice as shown in Figure 3B. These observations implicate the CXCL12/CXCR4 axis in LPS‐induced PTL.

### 3.3 AMD3100 suppressed the infiltration of immune cells stimulated by LPS

**FIGURE 4:**
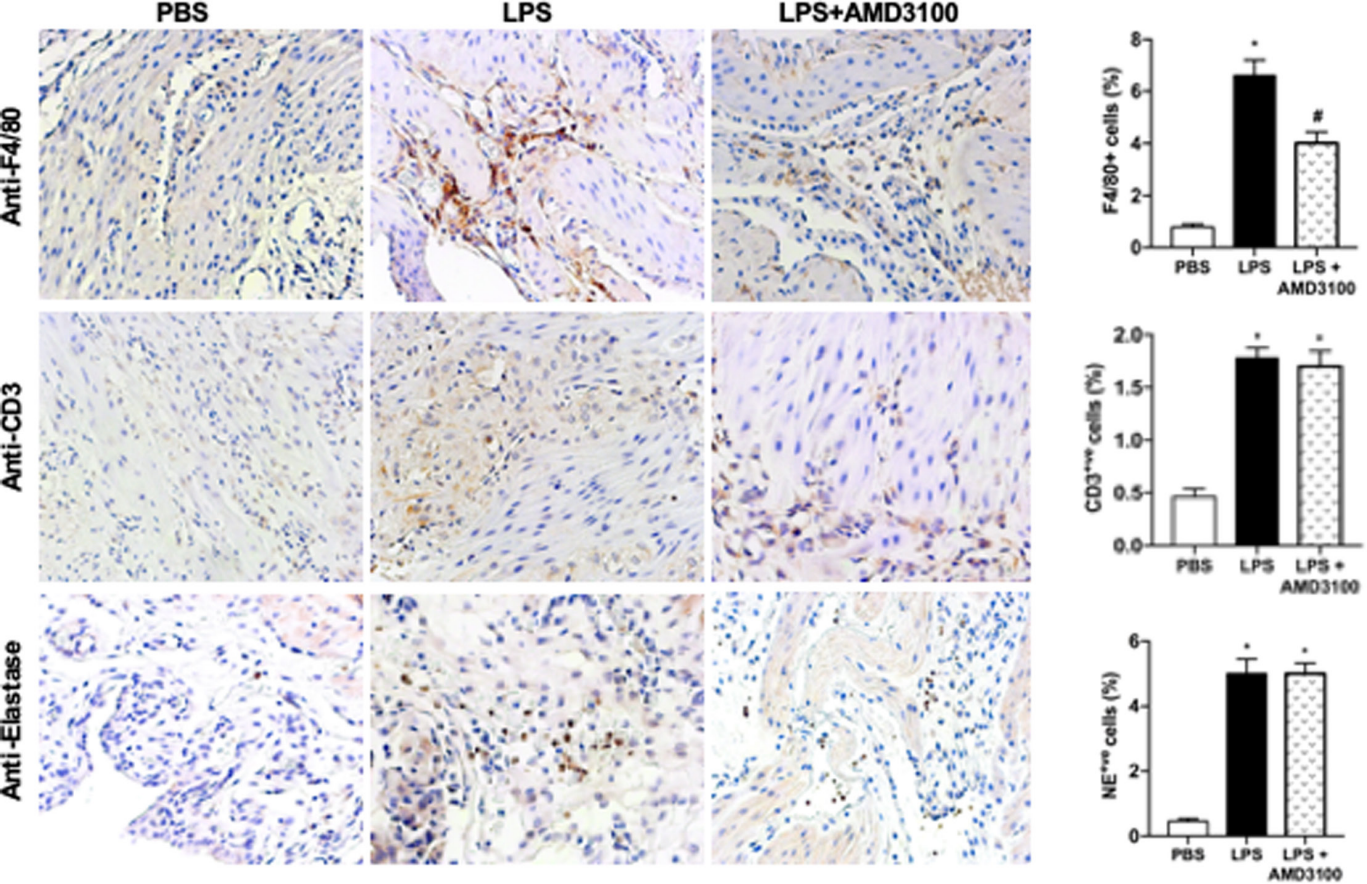
LPS‐stimulated engraftment of immune cells into the uterus while AMD3100 inhibited macrophage engraftment. Representative IHC images showing that the number of immune cells engrafted were increased by LPS treatment compared to PBS treated controls. Macrophages (anti‐F4/80); T cells (anti‐CD3) and leukocytes (anti‐elastase) are all increased by LPS treatment, however, only macrophages are decreased after AMD3100 treatment. On the right quantitative analyses demonstrates the significant increase in F4/80^+ve^, CD3^+ve^ and elastase^+ve^ cells in LPS‐treated mice. AMD3100 inhibited the macrophage (F4/80^+ve^) engraftment induced by LPS. Results shown as mean ± SE. *p < 0.05 versus PBS while **^#^** *p* < 0.05 versus LPS. Original magnification 400×. IHC, immunohistochemistry; LPS, lipopolysaccharide; PBS, phosphate buffered saline; SE, standard error

Since CXCL12 was the chemokine upregulated after LPS treatment, we next explored its effect on the infiltration of leukocytes into murine uterus. Figure 4 demonstrates that LPS enhanced the infiltration of neutrophils, macrophages, and T cells compared with PBS treatment after 6 h. AMD3100 treatment significantly suppressed macrophage infiltration but not the infiltration of T‐cells and neutrophils (Figure 4). These observations suggested that AMD3100 may prevent LPS‐induced PTL by suppressing CXCL12 induced macrophage recruitment.

### 3.4 AMD3100 suppresses M1 macrophage polarization stimulated by LPS

**FIGURE 5:**
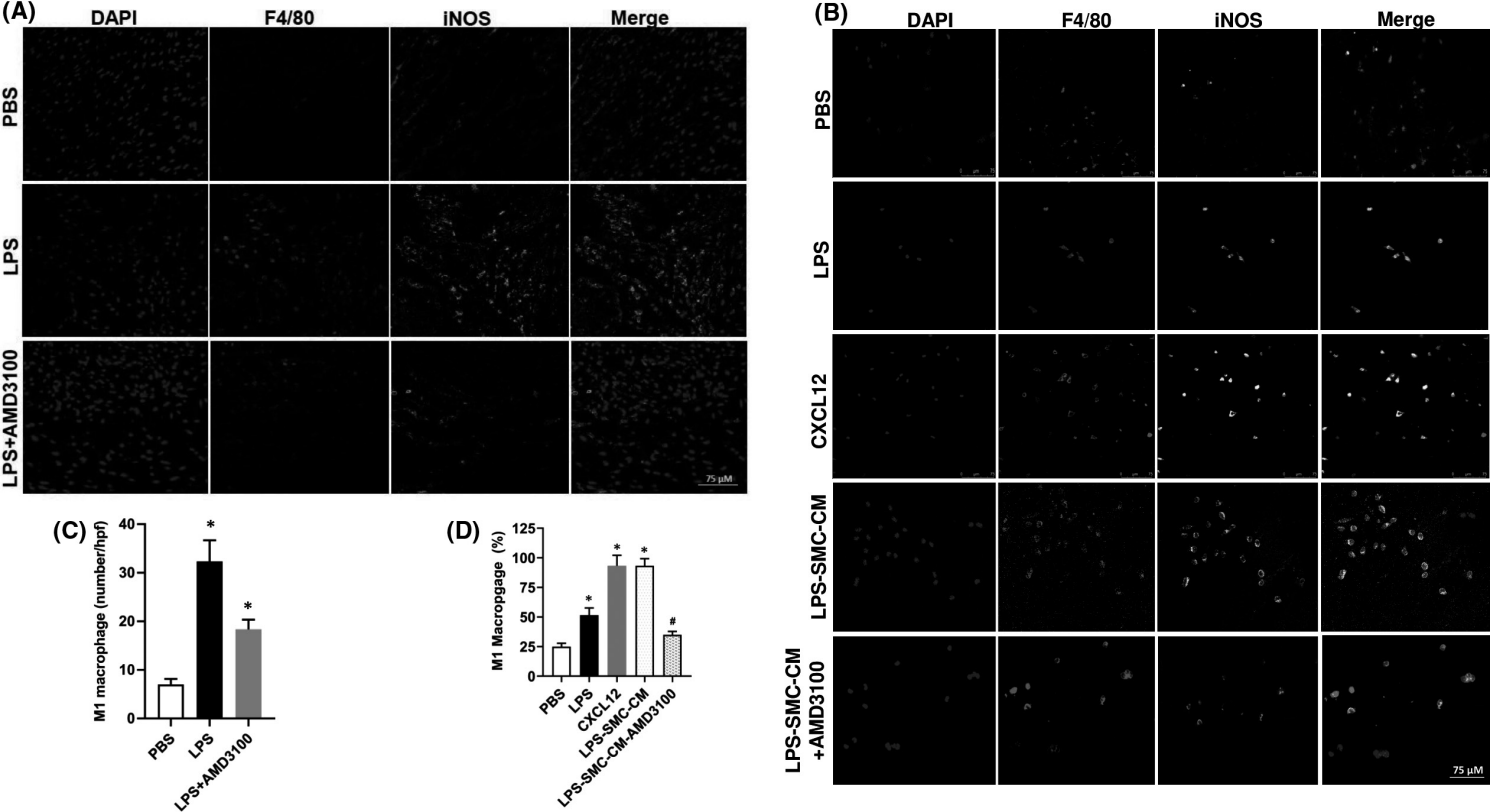
In vivo: AMD3100 inhibited LPS‐stimulated M1 macrophage polarization. (A, B) Fluorescence confocal microscopy analysis of M1 macrophage polarization. Representative images of IF showing DAPI, F4/80^+ve^, iNOS^+ve^ and merged images. Nuclei were stained by DAPI and are shown in blue. F4/80 marks macrophages while iNOS is a marker of M1 macrophages. (A) Uterus tissue from mice treated with PBS, LPS or LPS + AMD3100 respectively. (B) Macrophages treated with PBS, LPS, CXCL12, LPS‐SMC‐CM and LPS‐SMC‐CM + AMD3100 respectively. (C) Quantitative analyses of macrophage polarization (iNOS^+ve^ cells) in uterus showing significant increase in polarization in LPS‐treated macrophages compared to PBS. Treatment with AMD3100 inhibited the polarization stimulated by LPS. (D) Quantitative analyses of macrophage polarization (iNOS^+ve^ cells) in vitro showing significant increase in polarization in LPS, CXCL12 and LPS‐SMC‐CM treated macrophages compared with PBS treatment. AMD3100 inhibited the polarization stimulated by LPS. Results shown as mean ± SE. *p < 0.05 versus PBS while **^#^** *p* < 0.05 versus LPS‐SMC‐CM. Scale bar: 75 μm. DAPI, 4,6‐diamidino‐2‐phenylindole; IF, immunofluorescence; IHC, immunohistochemistry; LPS, lipopolysaccharide; PBS, phosphate buffered saline; SE, standard error; SMC‐CM, smooth muscle cell conditioned media

In order to investigate the M1 polarization of macrophages in the murine uterus, F4/80 and iNOS were evaluated by IF. Mice were injected with LPS and AMD3100. LPS‐induced macrophage M1 polarization (from 25% to 75% double positive cells), while AMD 3100 suppressed M1 macrophage polarization as shown in Figure 5A,C. To verify that uterine SMC production of CXCL12 could induce macrophage M1 polarization, macrophages were incubated with LPS‐stimulated uterine smooth muscle cell conditioned media (USMCs‐CM), with or without AMD 3100. As shown in Figure 5B,D, M1 macrophages (F4/80 and iNOS double positive) were polarized by LPS‐stimulated SMC‐CM (95.4%), CXCL12 (94%) or LPS (95.3%), compared with only 25% by PBS. However, M1 polarization was reduced to only 30% by a combination of LPS‐stimulated USMCs‐CM + AMD3100. These results suggested that LPS‐stimulated USMCs‐CM could induce macrophage M1 polarization, and this process is inhibited by AMD 3100 revealing that macrophage M1 polarization occurs through CXCL12/CXCR4 signalling pathway.

### 3.5 CXCR4 expression in macrophages

We determined the expression of CXCR4 in macrophages cultured in vitro. Figure S1 demonstrates that the number CXCR4^+ve^ macrophages were significantly decreased (1.5‐fold) by LPS treatment compared with PBS.

### 3.6 USMCs‐CM induced macrophage migration through CXCL12/CXCR4 signalling

**FIGURE 6:**
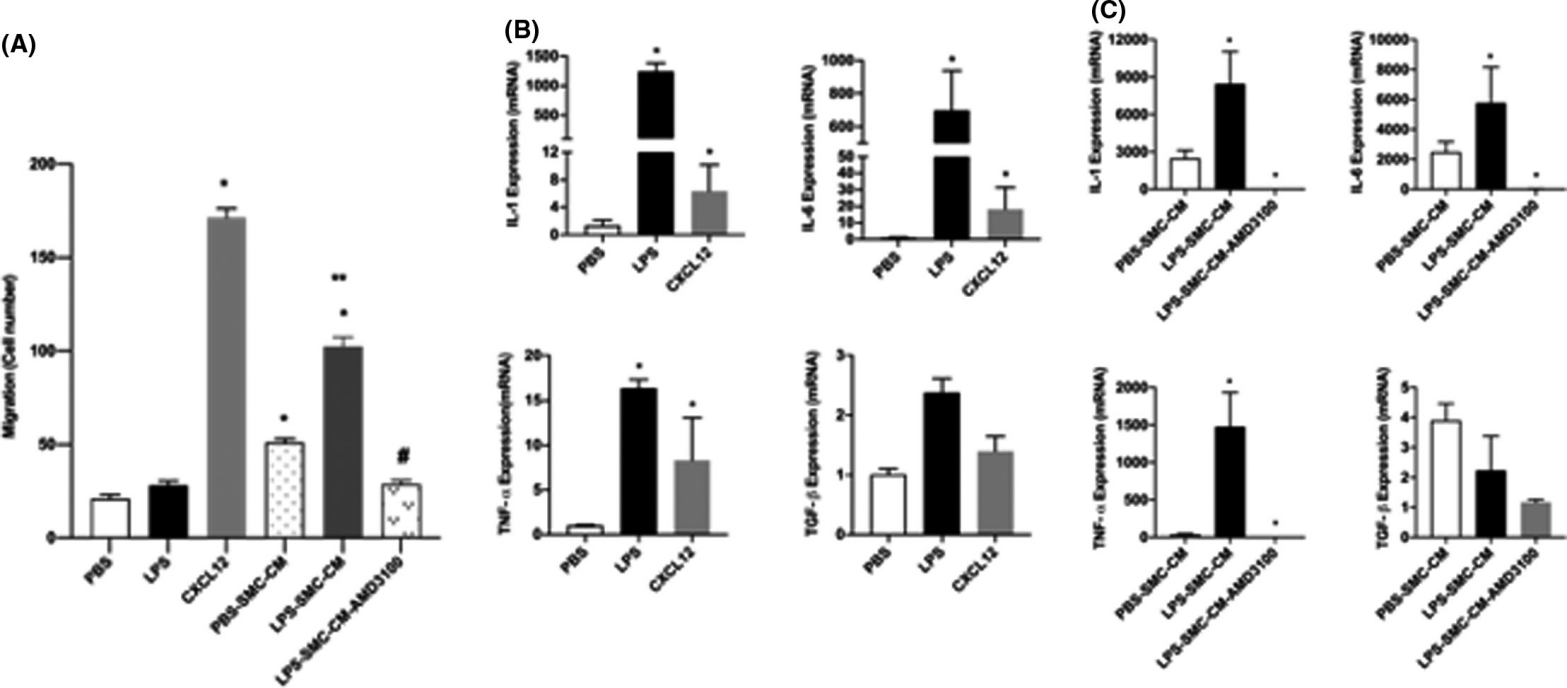
AMD3100 inhibited USMCs‐CM induced macrophage migration through CXCL12/CXCR4 signalling. (A) Macrophage attraction was determined by migration assay. Macrophages migrated significantly when treated with CXCL12 but not LPS. Conditioned medium from SMCs increased migration. Conditioned media from SMCs treated with LPS further increased migration. AMD3100 significantly inhibited macrophage migration stimulated by LPS‐treated SMC‐conditioned medium. Data are shown as a number of macrophages migrated. Each bar represents the mean ± SEM for data from two individual experiments and each experiment was performed in triplicate. *p < 0.05 versus PBS, **p < 0.05 versus PBS‐SMC‐CM and **^#^** *p* < 0.05 versus LPS‐SMC‐CM. (B, C) AMD3100 inhibited LPS stimulation of proinflammatory cytokines in M1 macrophage. (B) qRT‐PCR results showing significant increase in mRNA levels of IL‐1, IL‐6, TNF‐α induced by LPS or CXCL12 treatments compared with PBS. (C) qRT‐PCR results showing significant induction of IL‐1, IL‐6, TNF‐α expression stimulated by conditioned medium from smooth muscle cells treated with LPS. The effect of conditioned media on cytokine induction is blocked by AMD3100. Each bar represents the mean ± SEM for data from three individual experiments, and each experiment was performed in duplicate. *p < 0.05 versus PBS or PBS‐SMC‐CM. IHC, immunohistochemistry; IL, inyterleukin; LPS, lipopolysaccharide; PBS, phosphate buffered saline; SEM, standard error of mean; SMC‐CM, smooth muscle cell conditioned media; qRT‐PCR, quantitative real‐time polymerase chain reaction; TNF‐α, tumor necrosis factor alpha; USMC‐CM, uterine smooth muscle cell conditioned media

To determine if CXCL12 increases macrophage levels by chemotaxis to the uterus, we assayed the effect of CXCL12 on macrophage migration using a migration assay. As shown in Figure 6A, more macrophages migrated after CXCL12 or LPS‐USMCs‐CM treatment. When macrophages were treated with AMD3100 macrophage migration was inhibited significantly by 3.5 and 7.5‐fold compared with LPS‐USMCs‐CM and CXCL12 respectively. Notably, LPS alone did not directly induce macrophage migration. These results suggested that USMCs‐CM can induce macrophage migration through CXCL12/CXCR4 pathway.

### 3.7 SMC‐CM induce macrophage function through CXCL12/CXCR4 signalling

Multiple cytokines, including IL‐6, IL‐1 and TNF‐α, contribute to PTL. In particular, M1 macrophages produce proinflammatory cytokines such as IL‐1, IL‐6, IL‐12, IL‐23 and TNF‐α. As shown in Figure 6B, LPS‐stimulated macrophage expression of IL‐1, IL‐6 and TNF‐α mRNA (1400, 800 and 17‐fold, respectively, compared with PBS). Furthermore, CXCL12 similarly increased the expression of the same inflammatory cytokines (100, 150 and 8‐fold respectively). To investigate whether conditioned medium collected from SMC (SMC‐CM) cultured in vitro can stimulate macrophages to secrete PTL‐related cytokines through CXCL12/CXCR4 signalling, macrophages were incubated in SMC‐CM media or LPS‐SMC‐CM with or without AMD3100 as shown in Figure 6C. In the LPS‐SMC‐CM group, mRNA levels of IL‐6, IL‐1 and TNF‐α RNA were significantly higher than the PBS conditioned group. AMD3100 suppressed mRNA levels significantly after stimulation by LPS. These results indicated that SMC‐CM can stimulate macrophages to secrete IL‐1, IL‐6 and TNF‐α through the CXCL12/CXCR4 signalling pathway.

## 4 DISCUSSION

Preterm birth remains a major healthcare burden worldwide and every year about 15 million babies are born prematurely, mostly due to infection induced by vaginal pathogens.^1^ Though it is well known that infection is an important cause of PTL, the molecular mechanisms responsible for PTL are still incompletely characterized. The importance of the cytokines and acute inflammation in PTL is established. The interaction of chemokine ligand CXCL12 with its chemokine receptor CXCR4 is an important event in normal pregnancy physiology but poorly understood in PTL. To address the infection related PTL, several mouse models have been developed using bacteria directly (*E. coli*) or LPS, the toxic component on the surface of gram‐negative bacteria. LPS induces PTL and preterm birth (PTB).^41^, ^42^, ^43^, ^44^, ^45^, ^46^, ^47^, ^48^ Treatment of mice with LPS is widely used to mimic human PTL. Thus, in this study, we used bacterial LPS to induce PTL in an established model that mimics infection; whether this also replicates other inflammatory events that lead to PTL remains to be investigated. Using a combination of in vivo and in vitro approaches, we demonstrated direct signalling between SMCs and macrophages mediated through CXCL12/CXCR4. We revealed that SMC‐derived CXCL12 can directly recruit macrophages, and induce macrophage M1 polarization, as well as regulate macrophage function that led to PTL.

In order to investigate whether the blockade of CXCL12‐CXCR4 signalling could prevent infection‐induced PTL, AMD3100 was injected into pregnant mice before LPS was injected. The administration of the CXCR4 inhibitor AMD3100 effectively suppressed LPS‐induced PTL. Collectively, the CXCL12/CXCR4 axis can have important pathophysiological roles in infection‐induced PTL. Targeting CLXL12/ CXCR4 signalling with agents such as AMD3100 has clinical promise for the prevention or treatment of PTL. Currently, progesterone is the only medical therapy for prevention in patients at high risk of preterm birth, and its efficacy has been questioned.^49^, ^50^ Furthermore, while antibiotics alone have not been demonstrated to stop the progression of PTL, we hypothesize that combining antibiotics with an inhibitor targeting a specific chemokine pathway implicated in preterm birth may have improved efficacy. Alternatively, use of an inhibitor such as AMD3100 in women with early PTL and no evidence of intraamniotic infection by amniocentesis may be a safe treatment strategy. Our mouse model most closely mimics infection‐induced inflammation, and it remains to be determined whether the same pathway is operative in inflammation leading to PTL in the absence of infection. These proposed approaches should be confirmed in further animal and clinical studies. Further other agents that block CXCL12 signalling may also prove efficacious and remain to be evaluated. Finally, monitoring of CXCL12 levels may also be useful in the evaluation of those at risk for PTL.

The level of CXCL12 in peripheral blood of mice increased with gestation and even further after LPS treatment, which implicated elevated‐CXCL12 in both term and also LPS‐induced PTL. These data correlate well with findings from human studies; Tseng et al. prospectively evaluated CXCL12 concentration in the amniotic fluid collected by amniocentesis in the second trimester of pregnancy.^35^ They found that pregnancies with higher levels of CXCL12 are more prone to preterm delivery, and their neonates had lower birth weights and 1 min Apgar scores. Aminzadeh et al. reported that the concentration of CXCL12 is elevated in umbilical vein serum and in the mother's serum after labor, including preterm delivery.^36^ However, there are no sequential studies of CXCL12 level during pregnancy in humans. In this study, we are the first to report the progressive increase in CXCL12 levels throughout the course of pregnancy in mice.

CXCL12 is produced by multiple different cell types. During pregnancy, several reports indicate that human first trimester trophoblasts and decidua stromal cells are the major sources of CXCL12.^51^ However, Red‐Horse et al. found that CXCL12 was rarely expressed in the STBs, particularly in second and third trimester pregnancies.^52^ In the present study, we observed CXCL12 mainly expressed in the murine myometrium, not in placenta or decidua. This suggested that myometrium is the main source of CXCL12 in late pregnancy in mice. The expression of CXCL12 usually depends on environmental factors, and hypoxia enhances trophoblasts expression of CXCL12.^53^, ^54^ LPS has also been reported to upregulate CXCL12 expression in cytotrophoblasts.^55^ This is consistent with our data that showed the LPS upregulated CXCL12 levels; however, in our model, the most significant increase was in SMC of the myometrium.

Labor, either term or preterm, resembles an inflammatory response that includes secretion of cytokines/chemokines by resident and infiltrating immune cells into myometrium.^56^ In this study, we observed that more immune cells infiltrated into myometrium, including macrophages, T cells and neutrophils after LPS injection, which is consistent with the study reported by Mizoguchi et al.^39^ Macrophages consistently reside in mouse myometrium but are increased immediately in LPS‐injected mice with labor onset.^57^ Macrophages are increased in the cervix and stroma in human PTL.^58^ Indeed, the systemic depletion of macrophages prevented LPS‐induced PTL in mice.^59^ Thus, macrophage infiltration may be associated with LPS‐induced PTL. In the present study, macrophage recruitment in myometrium was significantly suppressed by AMD3100; however, there was no significant difference in neutrophil and T‐cell numbers. In vitro, it was further confirmed that CXCL12 can induce macrophage migration; macrophages express various chemokine receptors including CXCR4, and impair macrophage recruitment in brain ischaemia^60^ and diabetic retinopathy,^61^ thereby preventing or alleviating pathological changes. In this study, CXCR4 was widely expressed by macrophages; 95% of macrophages were CXCR4 positive. These results demonstrate the essential involvement of the CXCL12‐CXCR4 interaction in macrophage recruitment in LPS‐induced PTL.

Macrophages can polarize into specific phenotypes and have specific biological functions in response to micro‐environmental stimuli. Macrophages have been classified as either M1 and M2 subtypes based on their activation states.^62^ M1 macrophages are functionally proinflammatory, while M2 macrophages are anti‐inflammatory.^63^, ^64^ Macrophages are polarized into M1 macrophages by LPS.^63^, ^64^ At the maternal–foetal interface, both the number and proportion of M1/M2 macrophages change throughout gestation to protect the foetus from the maternal immune microenvironment and establish foetomaternal tolerance. To sustain foetomaternal tolerance, more macrophages are polarized into alternatively activated (M2‐like) macrophages. However, in PTL, more classically activated (M1) macrophages have been observed at the maternal–foetal interface.^65^, ^66^ In the present study, CXCL12 induced macrophages M1 polarization, and AMD3100 inhibited this process. This suggested that the CXCL12/CXCR4 axis promotes PTL not only by regulating macrophage recruitment, but also by inducing macrophage MI polarization.

Macrophages are tissue‐resident immune cells that play a critical role in maintaining homeostasis and fighting infection. Besides phagocytosis and antigen presentation function, macrophages can secrete cytokines. M1 macrophages are able to produce proinflammatory cytokines, including IL‐1β, IL‐6 and TNF‐α, while M2 macrophages mainly secrete anti‐inflammatory mediators such as IL‐4, IL‐10 and TGF‐β.^67^ Several proinflammatory cytokines play an important role in the pathogenesis of PTL. For example, IL‐1 and TNF‐α administration induces PTL and genetic disruption of IL‐1 or TNF‐α receptors prevent LPS‐induced PTL.^68^, ^69^ Similarly, the inhibition of IL‐6 production prevented LPS‐induced PTL.^70^ In contrast, reduced level of transforming growth factor (TGF)‐β is a risk factor for PTL.^71^ In this study, IL‐1β, IL‐6 and TNF‐α RNA increased in macrophage after CXCL12 treatment while there was no change of TGF‐β. This suggested that CXCL12 can contribute to the production of proinflammatory cytokines by macrophages implicated in PTL.

The CXCR4–CXCL12 axis is also activated during tissue repair and remodelling. This function of the CXCR4–CXCL12 axis plays a prominent role in the recruitment of bone marrow‐derived progenitors to the uterus and endometrium.^21^, ^72^, ^73^ It is also operative in the repair and regeneration of multiple non‐reproductive tissues after damage.^74^, ^75^, ^76^, ^77^ While CXCL12 is essential in activating inflammatory pathways that lead to PTL, CXCL12 also activates an accompanying repair mechanism; the later involves recruitment of progenitors to repair damage after infection or other insult. Proper timing of CXCR4 inhibition in the treatment of PTL may need to include reversal after control of infection, thus reactivating recruitment of progenitor cells involved in tissue repair.

## 5 CONCLUSIONS

In summary, LPS treatment in pregnant mice‐induced PTL by increasing myometrial CXCL12, which recruits immune cells that in turn produce inflammatory cytokines. These effects were completely reversed by AMD3100 through blocking of CXCL12/CXCR4 signalling. Thus, the CXCL12/CXCR4 axis presents a promising target for preventing infection and inflammation‐related PTL.

## CONFLICT OF INTEREST

All authors declare no conflict of interest.

## AUTHOR CONTRIBUTIONS

**Lijuan Zhang:** Data curation (equal); Formal analysis (equal). **Ramanaiah Mamillapalli:** Data curation (equal); Formal analysis (equal); Methodology (equal); Project administration (equal); Software (equal); Supervision (equal); Visualization (equal); Writing – original draft (equal). **Shutaro Habata:** Data curation (equal). **Molly McAdow:** Visualization (equal). **Hugh S. Taylor:** Conceptualization (equal); Funding acquisition (equal); Investigation (equal); Supervision (equal); Validation (equal); Writing – review and editing (equal).

## Supporting information

Fig S1

## Data Availability

The data that support the above findings are available with the corresponding author.
