# Supplementary figures and images for: Myometrial‐derived CXCL12 promotes lipopolysaccharide induced preterm labour by regulating macrophage migration, polarization and function in mice
Source: J Cell Mol Med. 2022 Mar 23;26(9):2566–78. doi: 10.1111/jcmm.17252 (PMC9077289; doi:10.1111/jcmm.17252)

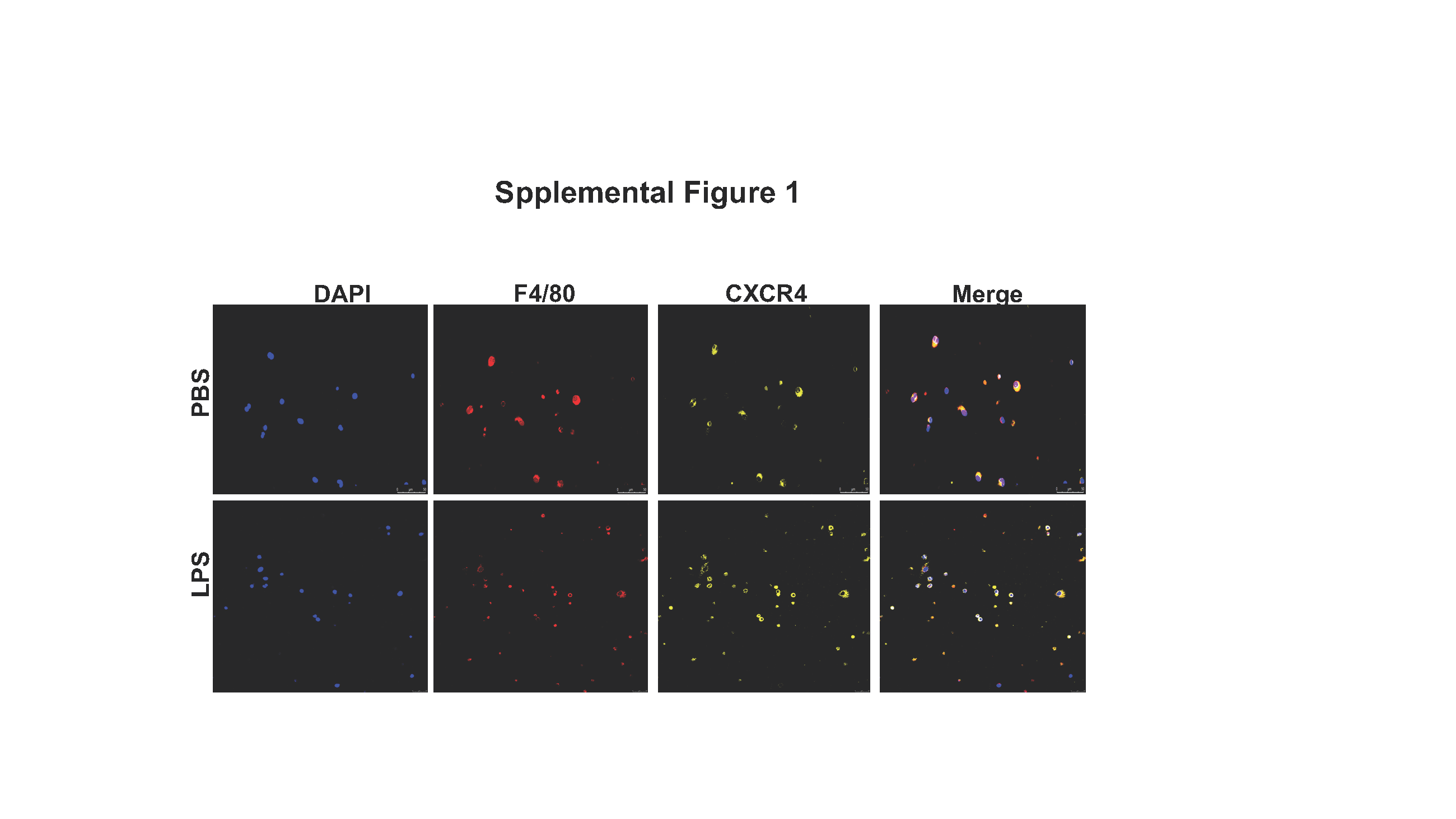

Supplement: Supplementary file 1 — Fig S1 [file JCMM-26-2566-s001.tiff]
